# Hypo-hydroxymethylation of rRNA genes in the precocious *Eriocheir sinensis* testes revealed using hMeDIP-seq

**DOI:** 10.1038/s41598-017-11517-1

**Published:** 2017-09-11

**Authors:** Genliang Li, Hui Qian

**Affiliations:** Youjiang Medical University for Nationalities, Baise, 533000 Guangxi The People’s Republic of China

## Abstract

Precocious puberty is a common phenomenon in crab breeding that seriously reduces the economic benefits for crab farmers. To address this problem, this study aimed to explore the potential functions of both methylation and hydroxymethylation of testis rRNA genes with respect to precocious puberty in *Eriocheir sinensis*. The results showed that the rRNA genes in normally developing testes of *E. sinensis* had low levels of methylation and high levels of hydroxymethylation; however, although methylation levels were similar, the level of hydroxymethylation in precocious testes was lower than normal. Highly significant differences (*P* < *0.01*) in the hydroxymethylation of the 18S and 28S rRNA genes were found between precocious and normal testes. Our results suggested that both the 18S and 28S rRNA genes, which are normally downregulated by hypo-hydroxymethylation, might be involved in the process of precocious puberty. Our results also implied that hydroxymethylation of the 18S and 28S rRNA genes might be used as an important epigenetic molecular marker to evaluate economically significant potential for growth and breeding in this species.

## Introduction

DNA methylation and hydroxymethylation are important epigenetic modifications for gene expression regulation. DNA methylation typically represses gene transcription, although it also plays other roles in the regulation of gene expression based on the different locations of methylcytosines in the gene. Hydroxymethylation, on the contrary, activates gene expression or prompts DNA demethylation. Both DNA methylation and hydroxymethylation are essential for the regulation of embryonic development, stem cell pluripotency, genomic imprinting, X-chromosome inactivation, and spermatogenesis^1–8^. Abnormal methylation or hydroxymethylation of DNA often causes a variety of diseases, such as cancer and male infertility^9–11^. Moreover, these abnormal DNA modifications may be inherited by offspring via the gametes, and the diseases these modifications cause could also be passed to future generations^12^.

The Chinese mitten crab *Eriocheir sinensis* is an important aquatic breeding species and has a very high economic value in China. This crab is used as a model for researching crustacean reproduction^13–20^. Precocious puberty is a common phenomenon in crab breeding that leads to small sizes in mature individuals and their descendants, seriously reducing the economic benefits of the crab farmers and hindering the development of crab aquaculture. Because DNA methylation and hydroxymethylation are associated with spermatogenesis and the development of the reproductive system, including the testis, and because precocious-puberty-related epigenetic markers could be passed to future generations via spermatozoa, we speculated that genomic DNA methylation and hydroxymethylation would be related to sexual precocity in this crab. The greater part of spermatozoal formation occurs during testis development. Therefore, an analysis of genomic methylation and hydroxymethylation in the developing testes of the crab, comparing the methylation and hydroxymethylation of the testis genome in precocious crabs with those of normal ones, might help us to understand the epigenetic molecular mechanisms of precocious puberty in *E. sinensis*.

Methylated DNA immunoprecipitation (MeDIP) and hydroxymethylated DNA immunoprecipitation (hMeDIP) can effectively enrich methylated and hydroxymethylated DNA fragments in genome-wide assays^21, 22^. Library construction, high-throughput sequencing and bioinformatic analysis of these methylated and hydroxymethylated DNA fragments can provide us maps of genome-wide methylation and hydroxymethylation under particular physiological or pathological conditions. These technologies are called methylated DNA immunoprecipitation sequencing (MeDIP-seq) and hydroxymethylated DNA immunoprecipitation sequencing (hMeDIP-seq)^23, 24^. However, because there is as yet no complete genome map of the crab, the genomic methylation and hydroxymethylation patterns of the crab cannot be completely analysed by mapping to the current genome draft of *E. sinensis*. In addition, ribosomal RNA (rRNA) genes are associated with the expression of many genes and physiological processes, including reproductive processes^25–27^. Therefore, using the technologies of MeDIP-seq and hMeDIP-seq and using the rRNA gene sequences of this species from the National Center for Biotechnology Information (NCBI) database as the genetic background, both methylation and hydroxymethylation of these rRNA genes in the testes of precocious and normal crabs were analysed and compared to assess abnormal DNA methylation and hydroxymethylation traits associated with precocious puberty in *E. sinensis*. The results provide basic data regarding the study of sexual precocity and determine the values of epigenetic methods in the identification of sexual precocity in *E. sinensis*.

## Results

### Data from MeDIP-seq and hMeDIP-seq

The data from the precocious and normal testis samples produced by MeDIP-seq and hMeDIP-seq using the Illumina HiSeq. 2500 and the analysis results of the software FastQC are shown in Supplementary Table S1. The results indicate that the data of MeDIP-seq were of high quality and met the standard for subsequent analysis.

### Low methylation and high hydroxymethylation of rRNA genes in developing testes

The results showed that there were 104,750 methylated clean reads and 373,676 hydroxymethylated clean reads that mapped to the rRNA genes in *E. sinensis*. Their percentages were 0.10% and 0.27% of the total clean reads, respectively. The methylation level of the rRNA genes was significantly lower than the hydroxymethylation level (*P* < *0.01*).

### Hypo-hydroxymethylation of total rRNA genes in precocious testes

**Figure 1:**
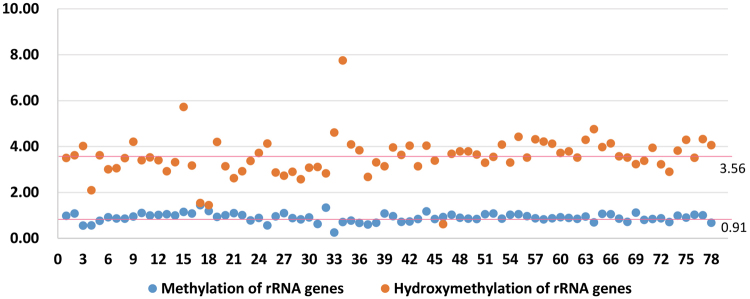
The ratios of methylation and hydroxymethylation of rRNA genes in normal and precocious testes of *E. sinensis*. The X axis represents the rRNA gene sequences, and the Y axis represents the ratios of methylated and hydroxymethylated clean reads mapped to the rRNA genes in normal and precocious testes. The rRNA genes with less than 50 total mapped clean reads in normal and precocious testes were ignored. The rRNA genes in the precocious testes were obviously hypo-hydroxymethylated compared to those in the normal testes.

**Figure 2:**
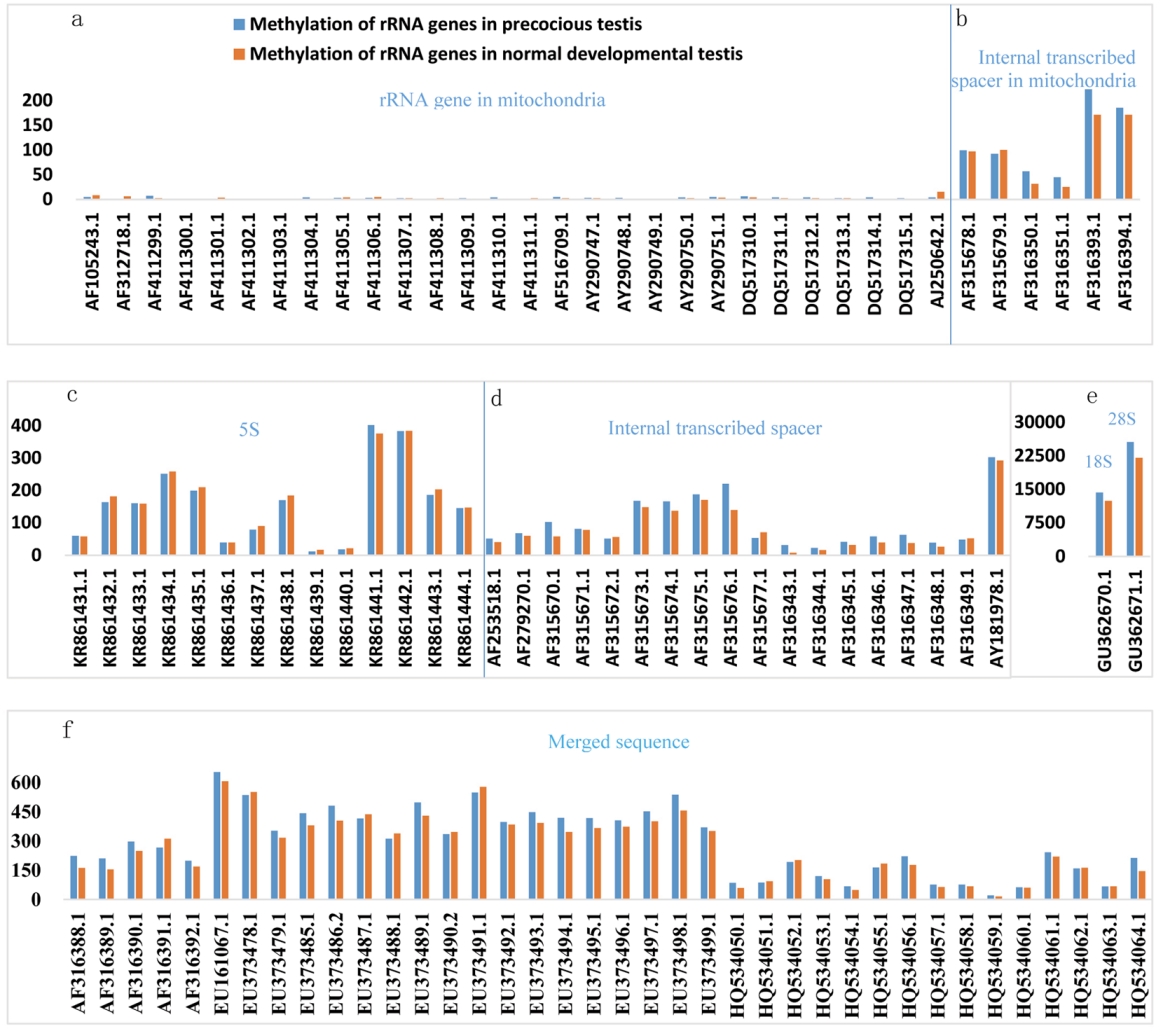
The level of methylation of the rRNA gene sequences in the precocious and normal testes. The X axis represents the rRNA gene sequences, and the Y axis represents the number of methylated clean reads mapped to the rRNA genes in the normal and precocious testes. (**a**) rRNA gene in mitochondria; (**b**) internal transcribed spacer in mitochondria; (**c**) 5S rRNA gene; (**d**) internal transcribed spacer; (**e**) 18S rRNA gene and 28S rRNA gene; and (**f**) merged sequence.

**Figure 3:**
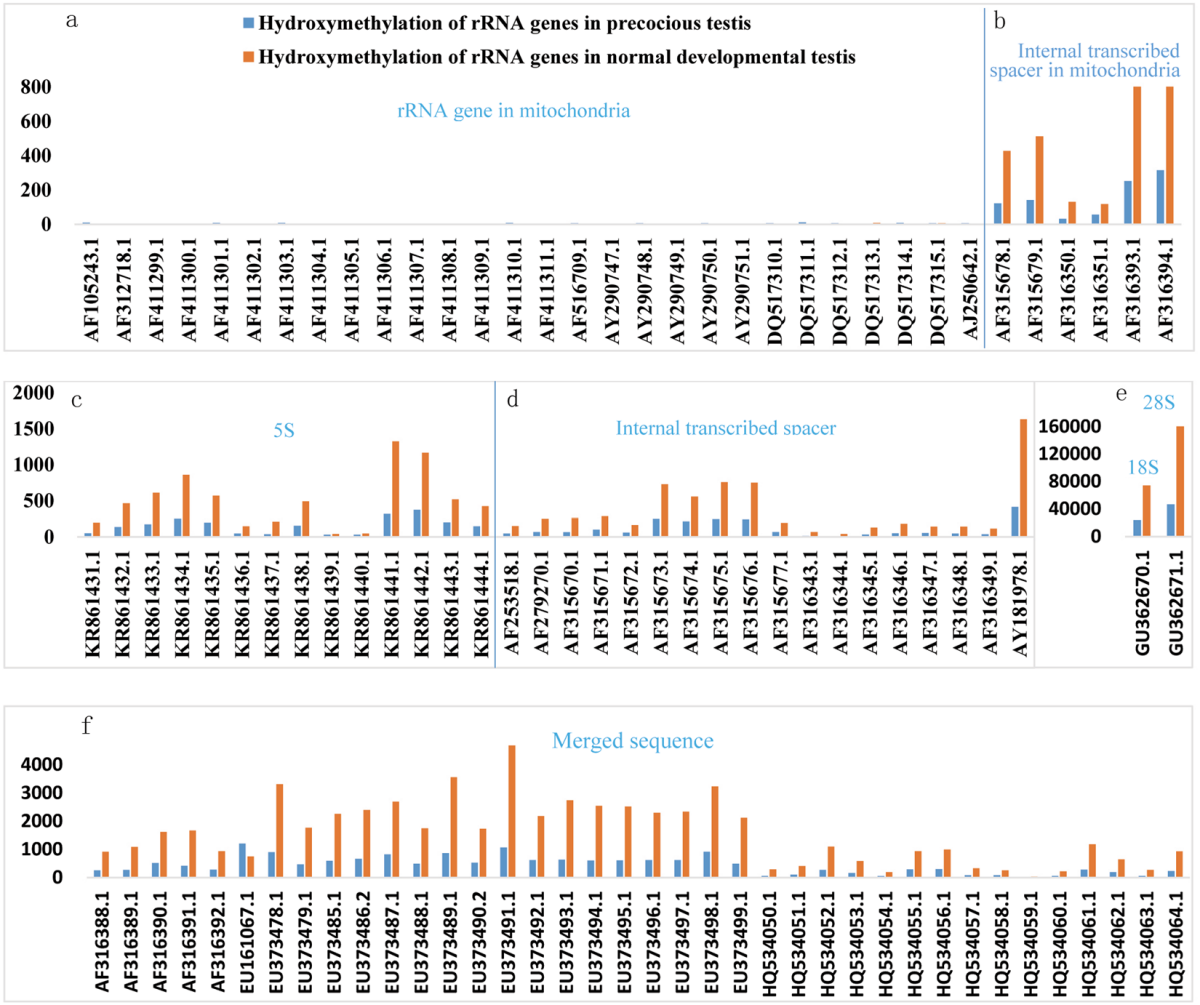
The level of hydroxymethylation of the rRNA gene sequences in the precocious and normal testes. The X axis represents the rRNA gene sequences, and the Y axis represents the number of hydroxymethylated clean reads mapped to the rRNA genes in the normal and precocious testes. (**a**) rRNA gene in mitochondria; (**b**) internal transcribed spacer in mitochondria; (**c**) 5S rRNA gene; (**d**) internal transcribed spacer; (**e**) 18S rRNA gene and 28S rRNA gene; and (**f**) merged sequence.

Only 56,281 and 48,469 methylated clean reads from the precocious and normal testis samples, respectively, mapped to the rRNA genes in *E. sinensis*. Their proportions of the total clean reads were 0.10% and 0.09%, respectively. However, the numbers of hydroxymethylated clean reads from the precocious and normal testis samples mapping to the rRNA genes in *E. sinensis* were 105,377 and 268,299, respectively. Their percentages were 0.13% and 0.45% of the total clean reads, respectively. Our data showed that the number of hydroxymethylated rRNA gene sequences was 5.04 times that of methylated rRNA gene sequences in the normal testes of *E. sinensis*, while the proportion in the precocious testes was only 1.31. The data also showed that the level of methylation of the rRNA genes in the precocious testes was similar to that in the normal testes, with a ratio of precocious to normal of 1.14, while the level of hydroxymethylation of the rRNA genes in the precocious testes was lower than that in the normal testes, with a ratio of 0.30 (Figs 1, 2, and 3).

**Figure 4:**
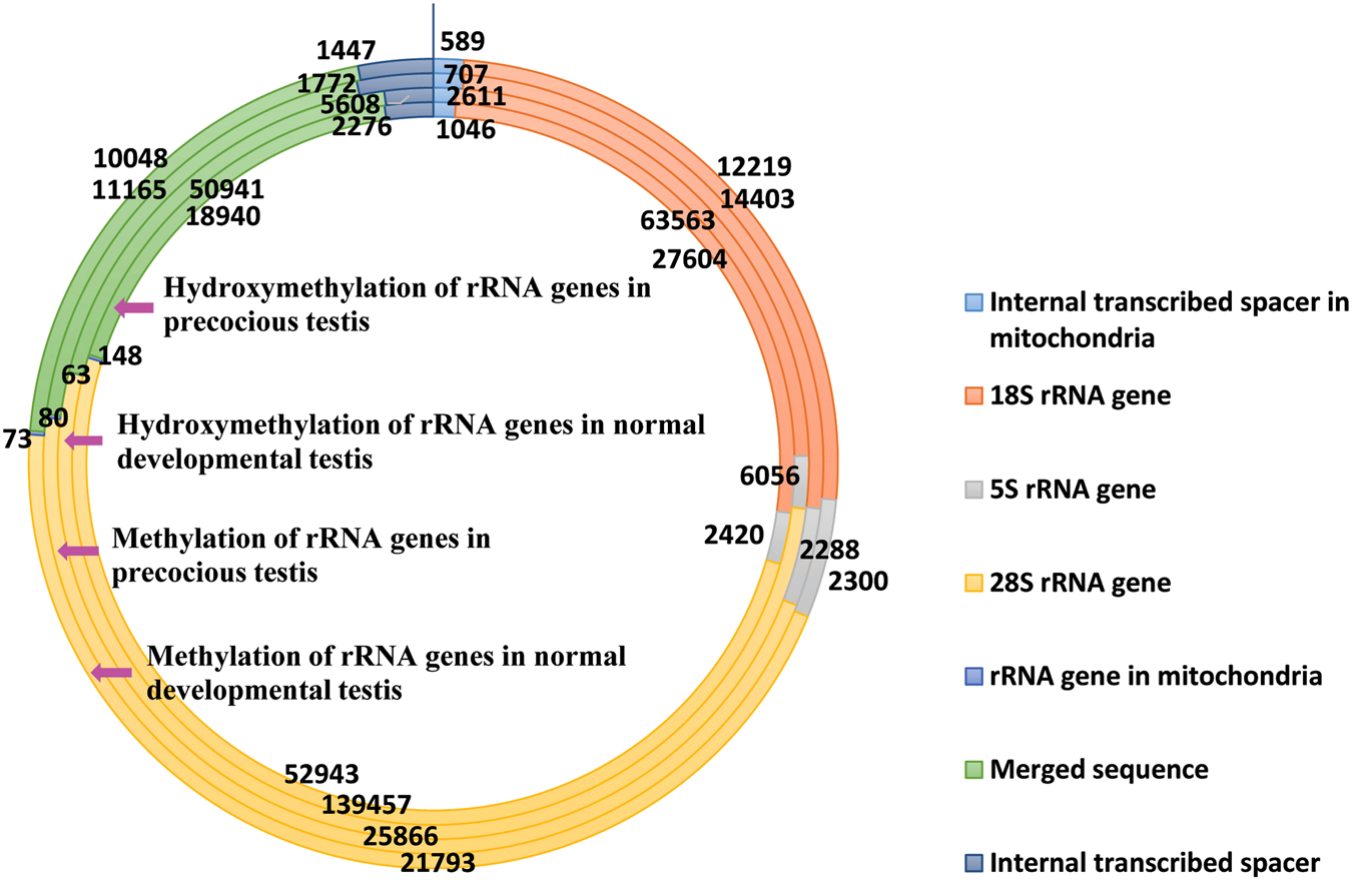
Distribution of methylated and hydroxymethylated rRNA gene sequences in the precocious and normal testes. The four circles represent, from inner to outer, the hydroxymethylation of rRNA genes in the precocious testis, the hydroxymethylation of rRNA genes in the normal developmental testis, the methylation of rRNA genes in the precocious testis, and the methylation of rRNA genes in the normal developmental testis.

We sorted the rRNA genes into seven types: 18S rRNA gene, 5S rRNA gene, 28S rRNA gene, internal transcribed spacer, merged sequence, rRNA gene in mitochondria, and internal transcribed spacer in mitochondria (Figs 2, 3, and 4). The “Merged sequence” category includes sequences including both an internal transcribed spacer and a partial sequence of an 18S, 5S, 28S, or 5.8S rRNA gene. There is no independent 5.8S rRNA gene of the crab in the NCBI database, and the 5.8S rRNA gene sequence was merged with its adjoining internal transcribed spacer.

The statistical analysis (chi-squared test) based on this classification showed that the genomic modifications of methylation and hydroxymethylation in the precocious testes of *E. sinensis* were highly significantly different from those in normal testes (*P* < *0.01*) and that there was a highly significant difference between the numbers of methylated and hydroxymethylated clean reads (*P* < *0.01*) (Table 1). The effect of hydroxymethylation on male precocious puberty was extremely significant (*P* < *0.01*) (Table 1), as well. However, for methylation, the ratios of methylated clean reads between precocious and normal testes mapping to individual rRNA genes or rRNA genes overall was between 0.5 and 2 (or the total number of clean reads mapped to an rRNA gene was less than 50). Therefore, the significance of rRNA gene methylation levels between precocious and normal testes was not further analysed.

**Table 1:** The effect of methylation and hydroxymethylation modifications of rRNA genes on male precocious puberty.

Factors of comparison	*df*	χ^2^	χ^2^_*0.05*_	χ^2^_*0.01*_
Precocious testes and normal ones^a^	18	3076.00^**^	28.87	34.81
Methylation and hydroxymethylation of rRNA genes^b^	6	2609.71^**^	12.59	16.81
Hydroxymethylation of rRNA genes^c^	6	461.70^**^	12.59	16.81

Notes: ^*^, Significant difference; ^**^, extremely significant difference. ^a^Comparison of methylation and hydroxymethylation modifications of rRNA genes between precocious and normal testes; ^b^comparison between the methylation and hydroxymethylation of rRNA genes; ^c^comparison of the hydroxymethylation of rRNA genes between precocious and normal testes.

### Hypo-hydroxymethylation of 18S and 28S rRNA genes in precocious testes

Our further analysis of the effects of various types of hydroxymethylated rRNA genes on the precocious puberty of the crab showed extremely significant differences (*P* < *0.01*) of hydroxymethylation of the 18S rRNA gene, the 28S rRNA gene, and the merged sequence between precocious and normal testes (Table 2). However, there appeared to be no significant difference in hydroxymethylation of the 5S rRNA gene, the internal transcribed spacer, or the internal transcribed spacer in mitochondria between the precocious and normal testes of the crab (*P* > 0.05). For the rRNA gene in mitochondria, the ratio of the total number of hydroxymethylated clean reads mapped to the rRNA genes in mitochondria between normal and precocious testes was more than 0.5 but less than 2, and the total number of hydroxymethylated clean reads in normal and precocious testes mapped to each rRNA gene in mitochondria was less than 50. Therefore, this difference of hydroxymethylation of rRNA genes in mitochondria might not be of significance, although its *P* value was less than 0.01.

**Table 2:** The effect of various types of hydroxymethylated rRNA genes on the precocious puberty of the crab (χ^2^_*1, 0.05*_ = 3.84, χ^2^_*1, 0.01*_ = 6.63).

Location	Precocious testes^a^	Normal testes^b^	Fold change^c^	χ^2^
18S rRNA gene	24187.06	74019.88	3.06	243.12^**^
5S rRNA gene	2120.44	7052.29	3.33	0.48
28S rRNA gene	46389.50	162399.36	3.50	86.00^**^
Internal transcribed spacer	1994.27	6530.58	3.28	1.64
Merged sequence	16595.53	59321.41	3.58	47.94^**^
rRNA gene in mitochondria	129.68	73.36	0.57	192.15^#^
Internal transcribed spacer in mitochondria	916.52	3040.54	3.32	0.26

Notes: ^*^, Significant difference; ^**^, extremely significant difference. ^#^The difference in hydroxymethylation of rRNA genes in mitochondria might not be of significance because the fold change was between 0.5 and 2. ^a^The number of hydroxymethylated clean reads mapped to the rRNA genes in precocious testes; ^b^the number of hydroxymethylated clean reads mapped to the rRNA genes in normal testes; ^c^the fold change of hydroxymethylated clean reads mapped to the rRNA genes in normal testes compared to that in precocious ones.

To understand the potentially different effects between the hydroxymethylation of the 18S rRNA gene, the 28S rRNA gene, and the merged sequence on precocious puberty in the crab, pairwise comparisons of the hydroxymethylated clean reads mapped to these three types of rRNA genes were carried out. The results showed that there were significant differences (*P* < *0.01 or P* < *0.05*) between the 18S rRNA gene, the 28S rRNA gene and the merged sequence, and each one was significantly different from the other rRNA genes, except for the internal transcribed spacer in mitochondria (Table 3).

**Table 3:** The effects of differing distributions of hydroxymethylation in the rRNA genes on the precocious puberty of the male crab (χ^2^_*1, 0.05*_ = 3.84 and χ^2^_*1, 0.01*_ = 6.63)

Location	χ^2^		
	18S rRNA gene	28S rRNA gene	Merged sequence
Internal transcribed spacer in mitochondria	0.095	1.944	3.646
5S rRNA gene	10.285^**^	4.047^*^	7.460^**^
rRNA gene in mitochondria	165.595^**^	200.945^**^	205.990^**^
Internal transcribed spacer	6.397^*^	6.468^*^	10.408^**^
Merged sequence	182.827^**^	4.127^*^	
28S rRNA gene	219.032^**^		

Notes: ^*^, Significant difference; ^**^, extremely significant difference.

The differences in hydroxymethylation of each merged sequence were further analysed between precocious and normal testes, because the differences in hydroxymethylation of the merged sequences as a whole were extremely significant between the two samples. The results showed that 17 of 38 merged sequences presented significant or extremely significant differences between precocious and normal testes (see Supplementary Table S2). Among them, 15 contained partial sequences of 28S or 18S rRNA genes. They were EU373478.1, EU373479.1, EU373485.1, EU373489.1, EU373491.1, EU373493.1, EU373494.1, EU373495.1, EU373496.1, EU373497.1, EU373499.1, HQ534050.1, HQ534052.1, HQ534061.1, and HQ534064.1. The other two contained partial sequences of 5.8S rRNA genes. They were AF316389.1 and AF316391.1.

### Verification of 18S and 28S rRNA gene hydroxymethylation using both RT-qPCR and gel electrophoresis

**Figure 5:**
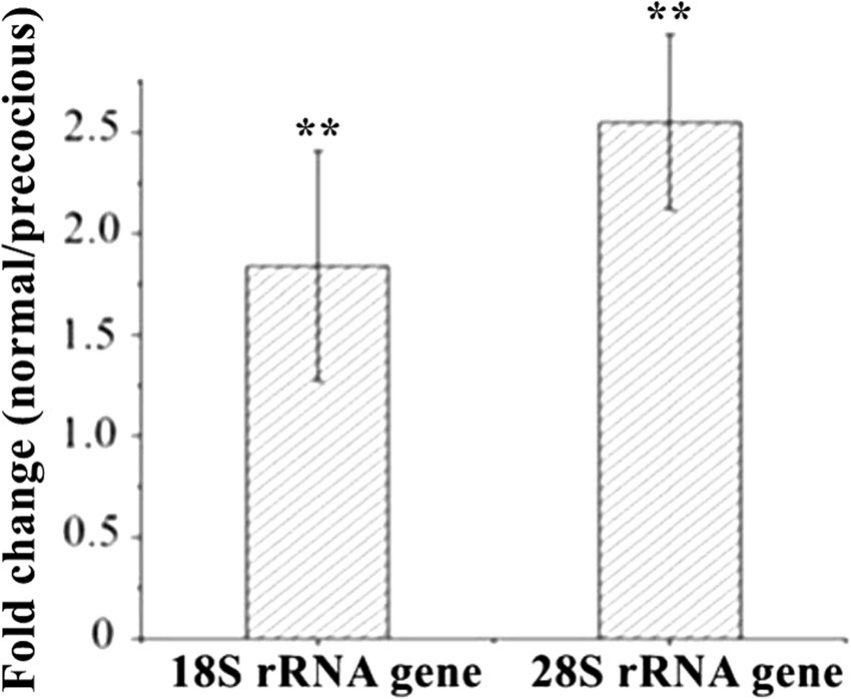
The validation of the expression levels of hydroxymethylated 18S and 28S rRNA genes in precocious and normal testes using RT-qPCR. The experiment was repeated three times. ** indicates a significant difference at *P* < *0.01*.

Both real-time quantitative polymerase chain reaction (RT-qPCR) and electrophoresis were used to validate the results of hMeDIP-seq related to the 18S and 28S rRNA genes. The primer sequences of RT-qPCR for the 18S and 28S rRNA genes were, for GU362670: GU362670F: TGATTACGTCCCTGCCCTT and GU362670R: ACATCTTTCCGTCAGCTCG; and for GU362671: GU362671F: ATCAGGTGGGGAGTTTGACTG and GU362671R: CCAGCATTTGTCCTTTCGCTC. The results were consistent with those of high-throughput sequencing (see Supplementary Fig. S1 and Fig. 5).

### Data deposition

The data of MeDIP-seq and hMeDIP-seq from the testis in *E. sinensis* were deposited in the NCBI database with accession numbers SRR5231463, SRR5231464, SRR5231465, and SRR5231466.

## Discussion

The ribosome is a factory where proteins are translated. Ribosomes are composed of both rRNAs and ribosomal proteins (rRNPs). The rRNAs account for approximately 60% of the total ribosome weight. In metabolically active cells, the number of ribosomes is significantly increased in order to meet the demand for proteins in cell metabolism. Therefore, the amount of rRNA or rRNP expression in a cell reflects the degree of metabolic activity. Furthermore, rRNA gene sequences can be used to identify and classify species^30–32^. They are regarded as an important basis for the diagnosis of disease caused by microbial infection^33, 34^. The rRNA genes are also associated with other physiological activities, such as gene expression and reproductive processes pertaining to asymmetric mitosis^35–38^. The expression of rRNA genes is regulated by DNA methylation and demethylation^35, 36^. Therefore, it is possible for an individual organism to control its gene expression and reproductive system development via the methylation and hydroxymethylation of its rRNA genes.

Our sequencing and analysis results showed that the rRNA genes in the developing testes of *E. sinensis* were methylated with a low frequency and hydroxymethylated with a high frequency, and the difference between methylation and hydroxymethylation was extremely significant (*P* < *0.01*). DNA methylation is usually associated with gene silencing, while DNA hydroxymethylation is related to the activation of gene expression^39, 40^. Therefore, our results showed that the rRNA genes were activated in the testes of the crab, and a large amount of rRNAs were synthesized. These rRNAs were involved in the assembly of a large number of ribosomes which were used to translate proteins. Therefore, the results indicated a high degree of protein synthesis in the developing testes of the crabs. It was consistent with the synthesis of a large number of male germ cells, namely, spermatogenesis, during the development of the testes. However, our results also showed that the level of hydroxymethylation of the rRNA genes in normal testes was obviously higher than that of their methylation (*P* < *0.01*), while the levels of hydroxymethylation and methylation of those genes were similar in the precocious testes. In addition, the level of methylation of rRNA genes in the precocious testes of *E. sinensis* was similar to that in the normal ones, while the level of hydroxymethylation of rRNA genes in the precocious testes was far lower than that in the normal ones (*P* < *0.01*). Those results indicated that protein synthesis was quantitatively different between the precocious testes and the normal ones. Due to the function of DNA hydroxymethylation in gene activation, the amount of rRNA gene expression in precocious individuals with hypo-hydroxymethylation of rRNA genes would be insufficient. This lack could result in relatively low ribosome content in precocious testes compared with that in normal testes. Puberty is the most important stage in the growth and development of various organs of the body. Downregulated expression of rRNA genes reduces the supply of proteins necessary for organ development, which not only reduces the size of the entire body but also hinders the functions of various organs, such as spermatogenesis, stress resistance, immunity, and so on. It could illuminate why the precocious testes of the crab are obviously smaller than normal in adults, why the spermatozoa count in precocious testes is dramatically less than that in normal ones, and why the mortality rate of precocious individuals is higher than that of normal ones. Because precocious individuals are distinctly smaller than normal individuals when mature, we speculated that the rRNA genes in other tissues of the precocious crab were also hypo-hydroxymethylated, resulting in downregulated expression *in vivo* of rRNA genes and an inadequate supply of proteins. Therefore, crabs with precocious puberty appear smaller than normal ones. This hypothesis requires further study to be validated.

Further analysis of the effects of the hydroxymethylation of seven types of rRNA genes on precocious puberty showed that there were extremely significant differences (*P* < *0.01*) of the hydroxymethylation of three rRNA types between precocious and normal testes: the 18S rRNA gene, the 28S rRNA gene, and the merged sequence.

Both methylation and hydroxymethylation of the rRNA genes in mitochondria were very rare compared to those of nuclear rRNA genes. This result is consistent with the low level of mitochondrial genome methylation and hydroxymethylation in this and other species^24, 41^. The CpG content in the rRNA genes in mitochondria was expected to be relatively lower than that in nuclear rRNA genes^24, 41^. In this study, we only considered rRNA genes for which the total numbers of methylated and hydroxymethylated clean reads were not less than 50 and the ratio of mapped hydroxymethylated clean reads between precocious and normal samples was not between 0.5 and 2, because genes which did not meet these criteria might affect the reliability of the statistical results. Therefore, the possible relationship of the hydroxymethylation of rRNA genes in mitochondria with precocious puberty was not further considered.

The results of the pairwise comparisons between the hydroxymethylation of the 18S rRNA gene, the 28S rRNA gene, and the merged sequence and those of the six other types of rRNA genes showed that there were significant differences (*P* < *0.01* or *P* < *0.05*) in hydroxymethylation between each of those three types of rRNA genes and the other types, except for the internal transcribed spacer in mitochondria. This result indicated that the effects of the hydroxymethylation of different types of rRNA genes on precocious puberty were different and that not all rRNA gene hydroxymethylation played a role in the process of precocious puberty. In particular, the hydroxymethylation of the 18S and 28S rRNA genes is likely involved in the physiological processes of sexual precocity in the crab, because the hydroxymethylation of 18S and 28S rRNA genes was significantly different between precocious and normal testes. Furthermore, the number of hydroxymethylated clean reads mapped to the 18S and 28S rRNA genes was the highest among all analysed rRNA genes. Therefore, the hydroxymethylation of the 18S and 28S rRNA genes might be used as an important molecular marker to distinguish precocious from normal crabs, as well as a reference to evaluate the economic significance of crab growth and breeding. The analysis of the significance of merged sequence hydroxymethylation further illuminated that hypo-hydroxymethylation of the 18S and 28S rRNA genes was associated with precocious puberty in the crab, because most of the significantly different merged sequences contained partial sequences of 28S or 18S rRNA genes. Therefore, we went on to verify the sequencing and analysis results for 18S and 28S rRNA gene hydroxymethylation using RT-qPCR and gel electrophoresis.

Hydroxymethylation plays a role in DNA demethylation in primordial germ cells^2, 3^ and is especially important in the self-renewal of stem cells, such as the pluripotent spermatogonial stem cells (SSCs), which can generate both new SSCs and spermatozoa. Significant and extremely significant differences in hydroxymethylation of the rRNA genes in the testes between precocious and normally developing crabs suggested that these hydroxymethylated rRNA gene sequences might be involved in the process of precocious puberty. Because DNA hydroxymethylation can affect the development of crabs due to the altered modification of these gene sequences, the abnormal hypo-hydroxymethylation of the rRNA gene sequences likely led to precocious puberty during crab development.

In conclusion, the rRNA genes in the developing testes of *E. sinensis* were methylated with a low frequency and hydroxymethylated with a high frequency, and the level of hydroxymethylation of rRNA genes in the precocious testes was significantly lower than that in normal testes, while the methylation of rRNA genes in precocious testes was similar to that in normal ones. In particular, there were extremely significant differences of hydroxymethylation of the 18S and 28S rRNA genes between crabs with precocious puberty and normally developing crabs. The hydroxymethylation of merged sequences between precocious and normal testes were extremely significantly different, and most of the significantly different merged sequences contained partial sequences of the 28S or 18S rRNA genes. Our results suggested that hypo-hydroxymethylated rRNA genes, especially the 18S and 28S rRNA genes, might be involved in the process of precocious puberty during the development of crab. Our results also hinted that the hydroxymethylation of the 18S and 28S rRNA genes might be used as an important molecular marker to distinguish precocious and normal crabs, as well as a reference to evaluate the economic significance of crab growth and breeding.

## Methods

### Animals and tissue preparation

Second instar male crabs (from the previous May to March) of 6–8 g were purchased from an aquaculture farm in Ganyu County, Jiangsu Province, China, in March 2015. They were divided into two groups consisting of precocious and normal crabs and held in wading pools; crabs were fed with potatoes and other food debris. Precocious crabs naturally appeared during the breeding process without intervention. The normal and precocious crabs are mainly distinguished by the characteristics of the chela, foot and gonad. Normal crabs have short, sparse hair on the propodus of the chela, but precocious crabs have fluffy, long, darker hair. The bristles on the pereopods of normal crabs are short and thin, while those of precocious crabs are long and thick. In the liver area, there are 2 regions of creamy white in precocious crabs but none in normal crabs.

For surgical removal of the testes, sixty of each group were placed at 0 °C for approximately 20 min for anaesthetization by hypothermia. The testes were immerged in DNA Guarder (Waryong, Beijing, China) preloaded in 1.5 ml Eppendorf tubes. The tubes were incubated at room temperature for 2 h so that the DNA Guarder could infiltrate into the tissues and then stored at −20 °C until required. For the use of *E. sinensis*, no approval is needed, because it is a common, economical, and edible invertebrate in China.

### Genomic DNA extraction, fragmentation, and MeDIP and hMeDIP

Genomic DNA was extracted from testis tissues using TIANamp Genomic DNA kits (TIANGEN, Beijing, China) according to the manufacturer’s instructions. DNA was fragmented by sonication. Ultrasound treatment was performed according to the previous method established in our laboratory^28^. Agarose gel electrophoresis was used to evaluate the quality of DNA fragmentation. The gel concentration was 1.5% (v/v). GelRed (Biotium, Fremont, CA, USA) staining was observed using a gel imaging system. The methylated and hydroxymethylated DNA fragments were captured using the MeDIP and hMeDIP kits^29^ according to their respective protocols. The quality of the captured DNA fragments was determined using an Agilent 2100 Bioanalyzer (Agilent 2100 expert High Sensitivity DNA Assay).

### Construction of sequencing library, sequencing and data analysis

The DNA library was constructed using the NGS Library Preparation Kit (KAPA Biosystems, USA) based on the manufacturer’s instructions. Double-ended sequencing (125 bp × 2) was performed using the Illumina2500 platform, and the data quality was evaluated according to conventional procedures^15^. Based on the draft genome and other nucleotide sequences relevant to the rRNA genes of the crab in the NCBI database, all rRNA gene-related sequences of the crab were downloaded and used to construct a reference sequence for the sequencing data. Methylated and hydroxymethylated clean reads were then harvested using a local BLAST database search.

### Analysis of methylation and hydroxymethylation of rRNA genes

The methylation and hydroxymethylation of rRNA genes in precocious and normal *E. sinensis* testes were analysed using Excel and SPSS 19.0 statistical software. The levels of methylation and hydroxymethylation of each gene sequence were compared based on the number of clean reads mapped to the rRNA genes of *E. sinensis*. The significance level of the statistical analysis was set at 0.05, and the ratio of the numbers of clean reads mapped to each gene sequence between the two groups of samples was conventionally not less than 2 or not more than 0.5. In the analysis of the data, the statistics were ignored when the number of total clean reads mapped to each gene were less than 50 in the two groups, because the number of clean reads was too small and might affect the reliability of the statistical results.

### Verification of hydroxymethylation of rRNA genes by RT-qPCR

According to the results of the analysis, the rRNA gene sequences with statistical significance were selected for verification using RT-qPCR and electrophoresis. RT-qPCR was performed according to the manufacturer’s instructions. The conditions of RT-qPCR reactions were: reaction mixture, 20 μL/tube; 95 °C for 8 min, 1 cycle; 95 °C for 10 s, 60 °C for 20 s, 72 °C for 10 s, 40 cycles; 72 °C for 3 min, 1 cycle. Primer-Blast software (https://www.ncbi.nlm.nih.gov/tools/primer-blast/) was used online to design the primers for RT-qPCR. The primers were synthesized by Genecore Inc. (Suzhou, China).

## Electronic supplementary material

Supplementary Dataset
