# Supplementary material for: Hypo-hydroxymethylation of rRNA genes in the precocious Eriocheir sinensis testes revealed using hMeDIP-seq
Source: Sci Rep. 2017 Sep 11;7:11237. doi: 10.1038/s41598-017-11517-1 (PMC5593943; doi:10.1038/s41598-017-11517-1)

**Supplementary Information**

**Hypo-hydroxymethylation of rRNA genes in the precocious Eriocheir sinensis testes revealed using hMeDIP-seq Supplementary files**

**Author list and affiliations:**

Genliang Li * & Hui Qian

Youjiang Medical University for Nationalities, Baise 533000, Guangxi, the People’s Republic of China

* Corresponding author, Youjiang Medical University for Nationalities, 98 Chengxiang Rd, 533000 Baise, the People’s Republic of China. E-mail: [ligenliang@163.com](mailto:ligenliang@163.com)

**Supplementary Table S1. Data of MeDIP-seq and hMeDIP-seq of precocious testes and normal ones in *E. sinensis***

|  |  | Precocious testes | Normal testes | Precocious testes | Normal testes |
| --- | --- | --- | --- | --- | --- |
| Total methylated raw reads | Number of reads | 55,610,776 | 54,444,312 | 79,594,344 | 59,886,362 |
|  | Number of bases (bp) | 6,951,347,000 | 6,805,539,000 | 9,949,293,000 | 7,485,795,250 |
| Total methylated clean reads | Number of reads | 55,500,204 | 54,350,532 | 79,367,628 | 59,718,724 |
|  | Number of bases (bp) | 6,292,945,524 | 6,216,647,066 | 8,880,337,985 | 6,600,163,320 |
|  | GC contents | 43.05% | 44.13% | 45.21% | 47.51% |
|  | Q30 percentage | 97.05% | 97.33% | 96.23% | 96.20% |

**Supplementary Table S2**. The difference of hypermethylation of each merging sequence between the precocious testes and normal ones.

| ID | Precocious testis ^a^ | Normal testis ^b^ | Fold change ^c^ | *X^2^* |
| --- | --- | --- | --- | --- |
| AF316392.1 | 275.132 | 931.610 | 0.295 | 0.001 |
| HQ534056.1 | 292.656 | 989.835 | 0.296 | 0.001 |
| HQ534054.1 | 52.573 | 185.157 | 0.284 | 0.036 |
| HQ534059.1 | 9.638 | 27.948 | 0.345 | 0.056 |
| HQ534058.1 | 77.983 | 251.535 | 0.310 | 0.100 |
| HQ534062.1 | 183.129 | 643.975 | 0.284 | 0.188 |
| EU373490.2 | 523.101 | 1730.465 | 0.302 | 0.197 |
| HQ534053.1 | 161.224 | 576.433 | 0.280 | 0.355 |
| HQ534055.1 | 288.275 | 931.610 | 0.309 | 0.449 |
| EU373487.1 | 814.881 | 2684.200 | 0.304 | 0.457 |
| HQ534060.1 | 56.078 | 214.270 | 0.262 | 0.589 |
| EU373492.1 | 618.608 | 2178.802 | 0.284 | 0.789 |
| EU373488.1 | 488.928 | 1737.452 | 0.281 | 0.931 |
| AF316388.1 | 251.474 | 915.307 | 0.275 | 1.051 |
| EU373498.1 | 917.398 | 3229.192 | 0.284 | 1.156 |
| HQ534057.1 | 81.488 | 321.405 | 0.254 | 1.494 |
| HQ534051.1 | 101.641 | 404.086 | 0.252 | 2.113 |
| AF316390.1 | 515.215 | 1617.507 | 0.319 | 2.258 |
| HQ534063.1 | 61.335 | 265.509 | 0.231 | 3.039 |
| EU373486.2 | 656.286 | 2396.566 | 0.274 | 3.139 |
| HQ534050.1 | 60.459 | 287.634 | 0.210 | 6.017^*^ |
| HQ534064.1 | 226.940 | 922.294 | 0.246 | 6.411^*^ |
| EU373478.1 | 897.245 | 3302.556 | 0.272 | 5.299^*^ |
| EU373479.1 | 465.271 | 1764.236 | 0.264 | 5.019^*^ |
| EU373485.1 | 593.198 | 2249.837 | 0.264 | 6.466^*^ |
| EU373496.1 | 616.856 | 2294.089 | 0.269 | 4.571^*^ |
| EU161067.1 | 1204.797 | 749.946 | 1.607 | 1594.355^#^ |
| HQ534052.1 | 264.617 | 1095.806 | 0.241 | 9.196^**^ |
| HQ534061.1 | 274.256 | 1178.486 | 0.233 | 13.535^**^ |
| AF316389.1 | 267.246 | 1080.667 | 0.247 | 7.159^**^ |
| EU373489.1 | 870.082 | 3554.091 | 0.245 | 26.743^**^ |
| EU373491.1 | 1058.469 | 4689.490 | 0.226 | 68.608^**^ |
| EU373493.1 | 634.380 | 2738.932 | 0.232 | 33.075^**^ |
| AF316391.1 | 411.821 | 1664.088 | 0.247 | 11.051^**^ |
| EU373494.1 | 601.960 | 2539.801 | 0.237 | 25.525^**^ |
| EU373495.1 | 612.475 | 2524.662 | 0.243 | 20.624^**^ |
| EU373497.1 | 615.980 | 2336.011 | 0.264 | 6.709^**^ |
| EU373499.1 | 492.433 | 2115.918 | 0.233 | 24.542** |
| Total | 16595.529 | 59321.410 | 0.280 | 47.938^**^ |

Notes：* Means significant difference; ** Means extremely significant difference. ^#^ The difference of hydroxymethylation of this merging sequence EU161067.1 might not be of significance because the number fold of its hydroxymethylation genes in normal testes to that in precocious ones was more than 0.5 and less than 2. ^a^ The number of hydroxymethylated clean reads matched to the merging sequences in precocious testes; ^b^ The number of hydroxymethylated clean reads matched to the merging sequences in normal testes; ^c^ The fold change of hydroxymethylated clean reads matched to the merging sequences in precocious testes to that in normal ones.

**Supplementary Figure S1**. The validation of the expression levels of hydroxymethylated 18S and 28S rRNA genes in the precocious and normal testes using RT-qPCR.


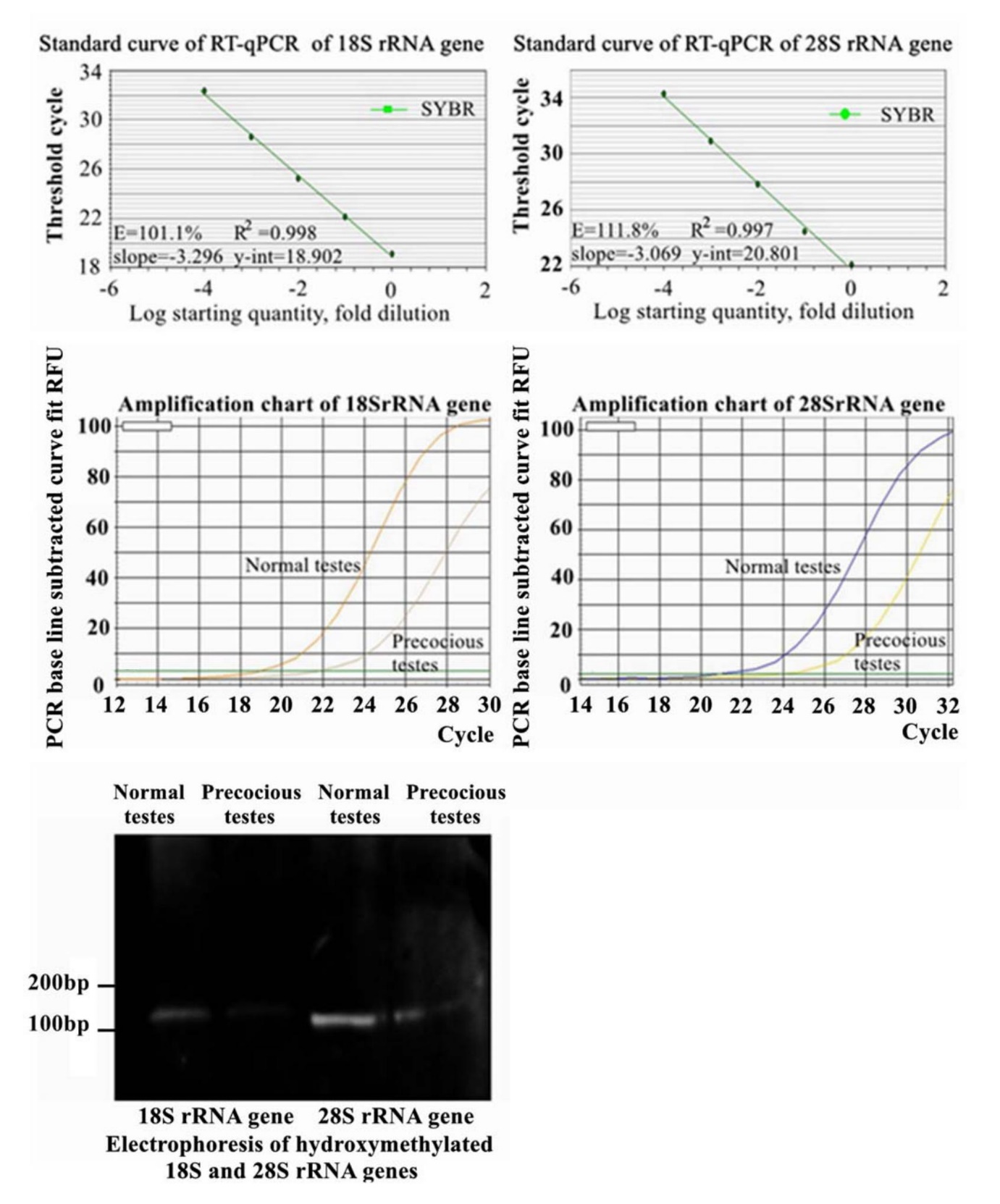

Supplement: Supplementary file 1 — Supplementary Dataset [file 41598_2017_11517_MOESM1_ESM.doc]
